# Automated bulk classification of glaucoma severity and progression using perimetry results

**DOI:** 10.1038/s41433-026-04847-w

**Published:** 2026-09-12

**Authors:** Nancy Wang, Arun J. Thirunavukarasu, Hita Patel, Rosia Li, Tasneem Z. Khatib

**Affiliations:** 1 University of Cambridge School of Clinical Medicine, University of Cambridge, Cambridge, UK; 2 Centre for Brain Repair, Department of Clinical Neurosciences, University of Cambridge, Cambridge, UK; 3 Department of Ophthalmology, Cambridge University Hospitals NHS Foundation Trust, Cambridge, UK; 4 International Centre for Eye Health, London School of Hygiene & Tropical Medicine, London, UK

**Keywords:** Medical research, Optic nerve diseases, Eye diseases, Health services, Health care

Ophthalmology is the busiest outpatient specialty with hundreds of thousands of NHS patients waiting for diagnosis or treatment, and an even greater number waiting for follow-up appointments [1]. Across the United Kingdom, glaucoma services are among the most under strain due to high prevalence of disease and suspicious clinical features, with a consequent requirement for longitudinal monitoring [2]. New approaches to tackling backlogs are needed, such as through pre-appointment triage to prioritise allocation of scarce clinic capacity. In one previous study, glaucoma doctors reviewing patients waiting for follow-up appointments were able to delay or discharge up to 40% of cases, and cancel up to 35% of planned visual field tests [3].

Clinical review requires expert appraisal of multimodal data from the clinical notes, imaging, and perimetry results. Perimetry data is key as it demonstrates whether glaucomatous loss of vision has occurred or is progressing, but is vulnerable to subjectivity. We developed the Glaucoma Field Defect Classifier (GFDC) to mitigate this issue by applying standardised criteria for grading the severity of glaucomatous visual field defects [4]. However, while useful for tasks such as identifying visual impairment [5], snapshot classification is not sufficient for triage as glaucoma pathways increasingly emphasise longitudinal risk-based assessment, accounting for different clinical trajectories [6].

We developed GFDC 2 to provide an automated, longitudinal assessment of perimetry data to accelerate triage workflow. GFDC 2 accepts batch exports of visual fields data at all time points from any number of patients, removing the requirement to manually input data. From these data, GFDC 2 computes the severity of any visual field defect by applying Hodapp-Parrish-Anderson criteria, whether there is central field involvement, and quantifies the rate of progression in terms of global metrics (e.g. mean deviation, visual field index) as well as at point-by-point level. To validate GFDC 2, we compared its output to data manually extracted by a masked researcher from individual perimetry plots as well as guided progression analysis (GPA) provided by FORUM software (Zeiss, Oberkochen, Germany). In a quality improvement project at Addenbrooke’s Hospital (PRN13551), the 35 patients (70 eyes) with the longest wait for a follow-up appointment were used to validate GFDC 2 in a triage-assistance role.

GFDC 2 exhibited close to perfect performance (Fig. 1). 100% agreement (intraclass correlation coefficient, ICC = 1.000) was reached in static parameters from the latest visual field test: mean deviation, visual field index, and pattern standard deviation. Absence or presence of a central visual field defect was correctly identified in all 70 cases (accuracy = 100.0%). For longitudinal parameters, close to 100% agreement was observed in mean deviation progression (ICC = 0.985) and visual field index progression (ICC = 0.994). Disagreement was infrequent, small in magnitude (Fig. 1), and in all cases was due to inclusion of unreliable fields in the proprietary GPA. Central defects were identified by GFDC in all 38 cases, and correctly excluded in all 32 negative cases.

**Fig. 1:**
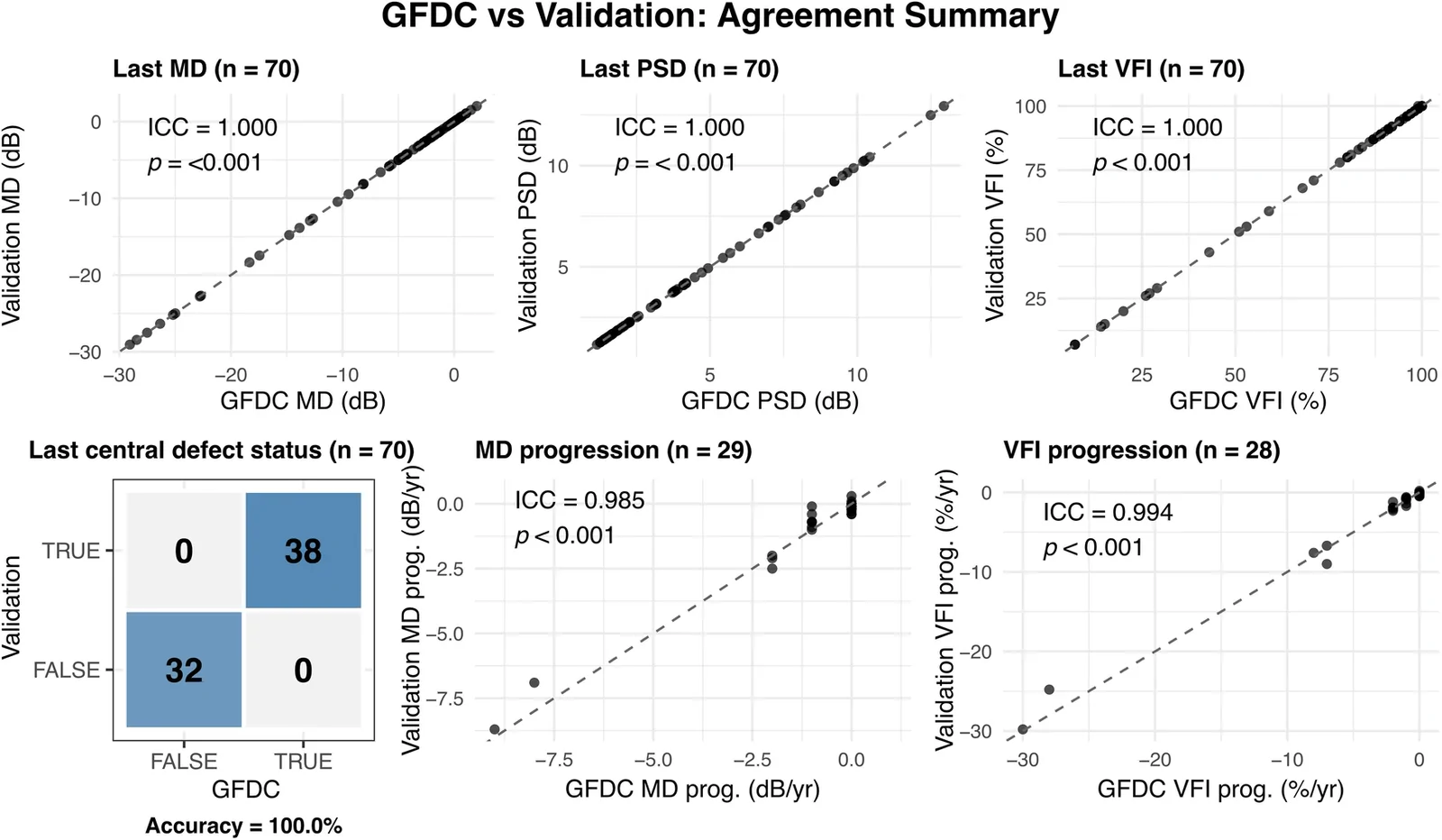
Agreement between the Glaucoma Field Defect Classifier (GFDC 2) and manual validation by a human researcher assisted by proprietary guided progression analysis (GPA) tools. For static parameters, agreement was perfect with intraclass correlation coefficients (ICC) of 1.000. Categorisation of central defects was also perfect, with no disagreement between GFDC 2 and manual judgements by a clinician. For progression metrics, agreement was close to perfect, with infrequent and small differences between GFDC and validation all due to unreliable perimetry results being included in GPA analysis.

GFDC 2 provides accurate and reliable perimetry interpretation based on the latest and longitudinal visual field results. With this tool, patients exhibiting central visual field involvement or rapid progression can be identified automatically and appropriately prioritised, supporting risk-stratified follow-up pathways as recommended in recent national glaucoma service guidance [6]. Automated interpretation of longitudinal perimetry may also facilitate asynchronous virtual review pathways by rapidly identifying patients requiring consultant or face-to-face review. Streamlining this process could reduce backlogs by up to 40% without requiring ophthalmologists to spend clinical time on onerous retrieval and interpretation of visual fields data [3].

## Data Availability

The datasets generated during the current study are not publicly available due to patient confidentiality, but details are available from the corresponding author on reasonable request.
